# Functional genomics indicates yeast requires Golgi/ER transport, chromatin remodeling, and DNA repair for low dose DMSO tolerance

**DOI:** 10.3389/fgene.2013.00154

**Published:** 2013-08-13

**Authors:** Brandon D. Gaytán, Alex V. Loguinov, Vanessa Y. De La Rosa, Jan-Michael Lerot, Chris D. Vulpe

**Affiliations:** Department of Nutritional Science and Toxicology, University of CaliforniaBerkeley, CA, USA

**Keywords:** DMSO, dimethyl sulfoxide, functional genomics, functional profiling, yeast, chromatin

## Abstract

Dimethyl sulfoxide (DMSO) is frequently utilized as a solvent in toxicological and pharmaceutical investigations. It is therefore important to establish the cellular and molecular targets of DMSO in order to differentiate its intrinsic effects from those elicited by a compound of interest. We performed a genome-wide functional screen in *Saccharomyces cerevisiae* to identify deletion mutants exhibiting sensitivity to 1% DMSO, a concentration standard to yeast chemical profiling studies. We report that mutants defective in Golgi/ER transport are sensitive to DMSO, including those lacking components of the conserved oligomeric Golgi (COG) complex. Moreover, strains deleted for members of the SWR1 histone exchange complex are hypersensitive to DMSO, with additional chromatin remodeling mutants displaying a range of growth defects. We also identify DNA repair genes important for DMSO tolerance. Finally, we demonstrate that overexpression of histone H2A.Z, which replaces chromatin-associated histone H2A in a SWR1-catalyzed reaction, confers resistance to DMSO. Many yeast genes described in this study have homologs in more complex organisms, and the data provided is applicable to future investigations into the cellular and molecular mechanisms of DMSO toxicity.

## Introduction

The dipolarity and low toxicity of dimethyl sulfoxide (DMSO) make it an unrivaled solvent in the field of toxicology. DMSO elicits numerous cellular effects, demonstrating the capacity to serve as a cryoprotectant, hydroxyl radical scavenger, and inducer of cellular differentiation and fusion (reviewed by Yu and Quinn, 1994). The pharmacological properties of DMSO have been documented in the treatment of brain edema, amyloidosis, rheumatoid arthritis, and schizophrenia, with infrequently reported systemic toxicities (Santos et al., 2003). The ubiquity of DMSO as a toxicant and drug solvent demands further identification of the cellular and molecular processes it may perturb, primarily to discern whether its effects influence those mediated by a compound of interest.

The unique genetic tools available in the model eukaryote *Saccharomyces cerevisiae* facilitate investigations into the cellular and molecular mechanisms of chemical resistance. The collection of barcoded yeast deletion mutants (Giaever et al., 2002) can be exploited to conduct functional genomic analyses (otherwise known as functional profiling) for a compound of interest. Pools of mutants are subjected to chemical treatment, and after DNA extraction, the strain-specific barcodes are amplified and hybridized to a microarray. Signal intensities correspond to strain numbers present in the pool after exposure, and indicate how the given insult alters the growth of individual mutants. With a high degree of conservation to more complex organisms (Steinmetz et al., 2002), yeast is an appealing model that can help identify human chemical susceptibility or resistance genes (Jo et al., 2009a; Blackman et al., 2012).

In this study, we utilized a genome-wide functional screen to identify yeast mutants exhibiting sensitivity to the common solvent DMSO. During preparation of this manuscript, a study was published implicating transcriptional control machinery and cell wall integrity as necessary for DMSO tolerance in *S. cerevisiae* (Zhang et al., 2013). Similarly, our results demonstrate that mutants lacking components of the SWR1 histone exchange complex exhibit hypersensitivity to DMSO. Here we corroborate and extend Zhang et al. (2013) by identifying additional SWR1 and conserved oligomeric Golgi (COG) complex members as required for DMSO resistance. We also provide extensive dose-response data for various deletion strains and present several novel DMSO-sensitive mutants. Finally, we indicate that overexpression of histone H2A.Z can confer DMSO resistance. Many yeast genes identified in this investigation have homologs that may contribute to DMSO response in more complex organisms.

## Materials and methods

### Yeast strains and culture

Functional profiling and confirmation analyses utilized the collection of BY4743 non-essential diploid yeast deletion strains (*MATa/MATα his3Δ1/his3Δ1 leu2Δ0/leu2Δ0 lys2Δ0/LYS2 MET15/met15Δ0 ura3Δ0/ura3Δ0*, Invitrogen). All assays were performed in liquid rich media (1% yeast extract, 2% peptone, 2% dextrose, YPD) at 30°C with shaking at 200 rpm, except overexpression experiments, which used liquid rich media containing 2% galactose and 2% raffinose (YPGal + Raf). For overexpression analyses, the *HTZ1* and *ARP6* HIP FlexGene expression vectors were transformed into strains of the BY4743 background.

### Functional profiling of the yeast genome and overenrichment analyses

Growth of the homozygous diploid deletion pools (4607 mutants in total), DNA extraction, PCR-amplification of strain barcodes, hybridization of Affymetrix TAG4 arrays, and differential strain sensitivity analysis (DSSA) were performed as described (Jo et al., 2009b). For DSSA, twelve 1% DMSO replicates were compared to 12 YPD replicates. Data files are available at the Gene Expression Omnibus (GEO) database. Significantly overrepresented Gene Ontology (GO) and MIPS (Munich Information Center for Protein Sequences) categories within the functional profiling data were identified with FunSpec (Robinson et al., 2002), using a *p*-value cutoff of 0.001 and Bonferroni correction.

### Growth curve and flow cytometry confirmation assays

Growth curve assays were performed as in North et al. (2011), with DMSO (VWR, #EM-MX1458-6) added to the desired final concentrations at a minimum two technical replicates per dose. Confirmation of growth defects by a flow cytometry based relative growth assay was performed as in Gaytán et al. (2013). Briefly, a culture containing GFP-tagged wild-type and untagged mutant cells was treated with DMSO, and a ratio of growth was calculated for untagged cells in treated versus untreated samples, as compared to the GFP strain. All graphs display the mean and standard error of three independent cultures. Three tests—regular *t*-test, Welch's test (*t*-test modification assuming unequal variances) and Wilcoxon Rank Sum (Mann–Whitney) test—were simultaneously applied to assess how possible violations of the assumptions underlying *t*-test (homoscedasticity and normality) affect statistical inference outcomes for the data. Raw *p*-values for each test statistic were corrected for multiplicity of comparisons using Benjamini–Hochberg correction. *P*-values indicated on graphs are derived from regular *t*-tests, with Welch and Wilcoxon Rank Sum test results (which are more robust but more conservative in terms of adjusted *p*-values) usually in agreement with regular *t*-tests (Table S1).

## Results

### Functional profiling in yeast identifies genes required for DMSO tolerance

Following growth of yeast homozygous diploid deletion mutant pools for 15 generations in 1% DMSO, DSSA identified 40 strains as sensitive to DMSO, as compared to YPD controls (Table 1; Table S2). To identify the biological attributes required for DMSO tolerance, enrichment analyses for the 40 sensitive strains was performed with FunSpec at a corrected *p*-value of 0.001. The COG complex, as well as its biological functions (cytoplasm to vacuole targeting pathway and intra-Golgi transport), were overrepresented in both GO and MIPS categories (Table 2).

**Table 1:** **Fitness scores for deletion strains identified as significantly sensitive to 1% DMSO during a 15 generation treatment**.

**ORF**	**Deleted gene**	**Log2 value 1% DMSO**	**Description of deleted gene**	**Confirmed**
YIL162W	*SUC2*	−4.54	Invertase, sucrose hydrolyzing enzyme	NS
YHR010W	*RPL27A*	−2.45	Component of the large (60S) ribosomal subunit	
YDR083W	*RRP8*	−2.41	Nucleolar protein involved in rRNA processing	S
YNL051W	*COG5*	−2.38	Component of conserved oligomeric Golgi complex; functions in protein trafficking	S
YER156C	-	−2.31	Putative protein of unknown function	
YOR304C-A	-	−2.26	Protein of unknown function	S
YML071C	*COG8*	−2.11	Component of conserved oligomeric Golgi complex; functions in protein trafficking	S
YLR371W	*ROM2*	−2.10	GDP/GTP exchange protein (GEP) for Rho1p and Rho2p	S
YJL132W	-	−2.07	Putative protein of unknown function	NS
YKR024C	*DBP7*	−1.93	Putative ATP-dependent RNA helicase; involved in ribosomal biogenesis	NS
YFR034C	*PHO4*	−1.91	Transcription factor of the myc-family; regulated by phosphate availability	NS
YNL107W	*YAF9*	−1.90	Subunit of NuA4 histone H4 acetyltransferase and SWR1 complex	S
YLR322W	*VPS65*	−1.83	Dubious ORF; overlaps the verified gene SFH1; deletion causes VPS defects	
YFR036W	*CDC26*	−1.65	Subunit of the Anaphase-Promoting Complex/Cyclosome (APC/C)	NS
YFR045W	-	−1.62	Putative mitochondrial transport protein	
YKR019C	*IRS4*	−1.61	Involved in regulating phosphatidylinositol 4,5-bisphosphate levels and autophagy	
YNL041C	*COG6*	−1.57	Component of conserved oligomeric Golgi complex; functions in protein trafficking	S
YLR261C	*VPS63*	−1.54	Dubious ORF; overlaps the verified gene YPT6; deletion causes VPS defects	
YBR227C	*MCX1*	−1.50	Mitochondrial matrix protein; putative ATP-binding chaperone	
YGL005C	*COG7*	−1.47	Component of conserved oligomeric Golgi complex; functions in protein trafficking	S
YJL205C	*NCE101*	−1.41	Protein of unknown function; involved in secretion of proteins	
YER032W	*FIR1*	−1.39	Involved in 3' mRNA processing	
YEL039C	*CYC7*	−1.36	Cytochrome c isoform 2	
YER110C	*KAP123*	−1.35	Karyopherin, mediates nuclear import of ribosomal proteins and histones H3/H4	S
YGL158W	*RCK1*	−1.35	Protein kinase involved in the response to oxidative stress	NS
YBR013C	-	−1.28	Putative protein of unknown function	
YGL031C	*RPL24A*	−1.26	Ribosomal protein L30 of the large (60S) ribosomal subunit	
YML116W	*ATR1*	−1.24	Multidrug efflux pump of the major facilitator superfamily	
YJR140C	*HIR3*	−1.22	Subunit of the HIR nucleosome assembly complex	S
YNL198C	-	−1.19	Dubious ORF unlikely to encode a protein	
YGL139W	*FLC3*	−1.14	Putative FAD transporter	
YGR089W	*NNF2*	−1.08	Interacts physically and genetically with Rpb8p (a subunit of RNA pols. I/II/III)	
YKL040C	*NFU1*	−1.06	Involved in iron metabolism in mitochondria	
YAL015C	*NTG1*	−1.05	DNA N-glycosylase and AP lyase involved in base excision repair	S
YGR108W	*CLB1*	−1.03	B-type cyclin involved in cell cycle progression	
YCR067C	*SED4*	−0.92	Integral endoplasmic reticulum membrane protein	
YIR001C	*SGN1*	−0.90	Cytoplasmic RNA-binding protein; may have a role in mRNA translation	
YDL211C	-	−0.88	Putative protein of unknown function; GFP-fusion protein localizes to vacuole	
YDR534C	*FIT1*	−0.88	Mannoprotein that is incorporated into the cell wall	
YER098W	*UBP9*	−0.87	Ubiquitin-specific protease that cleaves ubiquitin-protein fusions	

Fitness is defined as the normalized log2 ratio of strain growth in the presence vs. absence of DMSO. The confirmed column indicates whether the strain was confirmed as sensitive (S) or not sensitive (NS) by relative growth assays. Sensitivity is defined as a relative growth ratio of <0.9 in DMSO versus a wild-type GFP expressing strain.

**Table 2:** **MIPS or GO categories associated with genes required for DMSO resistance**.

	***p*-value**	**Genes identified**	***k***^**a**^	***f***^**b**^
**GO BIOLOGICAL PROCESS CATEGORY**				
Cytoplasm to vacuole targeting (CVT) pathway [GO:0032258]	2.38E–006	*COG7 IRS4 COG8 COG6 COG5*	5	37
Intra-Golgi vesicle-mediated transport [GO:0006891]	1.12E–005	*COG7 COG8 COG6 COG5*	4	24
**GO CELLULAR COMPONENT CATEGORY**				
Golgi transport complex [GO:0017119]	7.94E–008	*COG7 COG8 COG6 COG5*	4	8
Golgi membrane [GO:0000139]	6.43E–004	*SED4 COG7 COG8 COG6 COG5*	5	117
**MIPS FUNCTIONAL CLASSIFICATION CATEGORY**				
Intra Golgi transport [20.09.07.05]	4.16E–005	*COG7 COG8 COG6 COG5*	4	33

Strains exhibiting sensitivity to 1% DMSO, as identified by DSSA, were analyzed with FunSpec for overrepresented biological attributes.

a Number of genes in category identified as sensitive to DMSO.

b Number of genes in GO or MIPS category.

### Mutants defective in Golgi/ER transport are sensitive to DMSO

**Figure 1:**
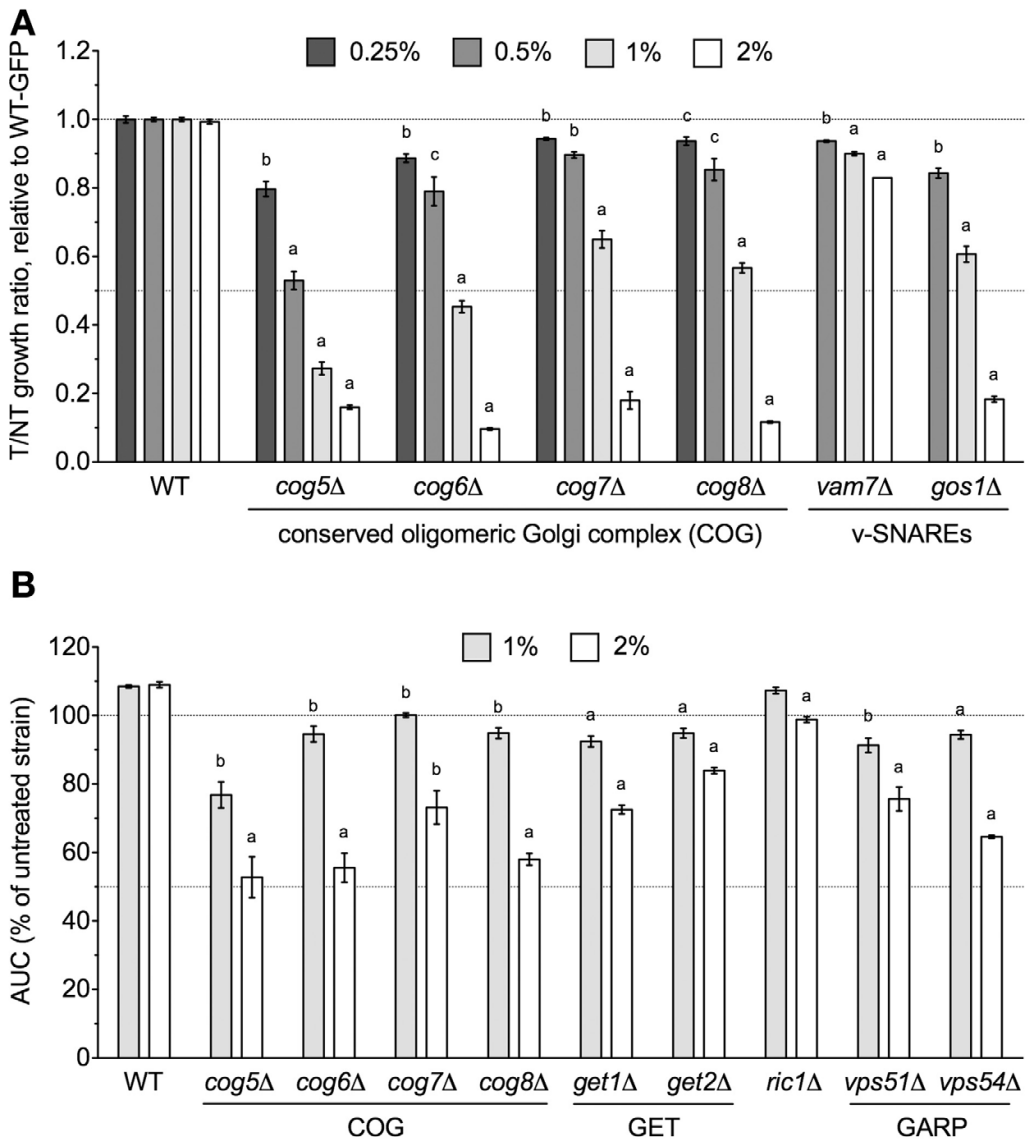
**Golgi/ER transport mutants are sensitive to DMSO**. Statistical significance between wild-type and mutant strains was calculated by *t*-test, where ^a^*p* < 0.001, ^b^*p* < 0.01, and ^c^*p* < 0.05. **(A)** Assessment of COG and vacuolar SNARE mutant growth in DMSO. Mutant strains were grown in competition with a GFP-expressing wild-type strain in the indicated DMSO concentrations and relative growth ratios (treatment vs. control) were obtained. The ratio means and standard errors are shown for three independent cultures. **(B)** Analysis of COG, GET, and Golgi-Associated Retrograde Protein (GARP) deletions in DMSO. Growth curves for three independent cultures were obtained for the indicated strains and doses of DMSO. The area under the curve (AUC) was calculated and is shown as a percentage of the untreated strain's AUC.

Overrepresentation analyses suggested that subunits of COG, a protein complex that mediates fusion of transport vesicles to Golgi compartments, were required for DMSO tolerance. Therefore, we performed relative growth assays in which the growth of COG deletion strains was compared to a wild-type GFP-expressing strain in various DMSO concentrations. Deletion of genes encoding any of the four non-essential subunits of COG (*COG5, COG6, COG7*, and *COG8*) resulted in dose-dependent sensitivity to DMSO, with statistically significant growth defects observed at DMSO concentrations as low as 0.25% (Figure 1A). Growth curve assays also confirmed sensitivity of the individual COG deletions under non-competitive conditions (Figure 1B). To identify additional sensitive Golgi/ER transport strains not present in the functional profiling data, we tested the DMSO sensitivity of various mutants displaying synthetic lethality or sickness with at least one COG gene. Analysis of relative growth by flow cytometry found that strains lacking vacuolar SNAREs (*vam7*Δ and *gos1*Δ) were DMSO-sensitive (Figure 1A). Growth curve experiments were performed as an alternative for strains demonstrating severe fitness defects in the relative growth assay, with mutants defective in retrograde Golgi transport (*ric1*Δ, *vps51*Δ, and *vps54*Δ) as well as those deleted for components of the Guided Entry of Tailanchored (GET) Golgi/ER trafficking complex (*get1*Δ and *get2*Δ) exhibiting dose-dependent DMSO sensitivity (Figure 1B).

### Chromatin remodeling machinery is required for DMSO tolerance

**Figure 2:**
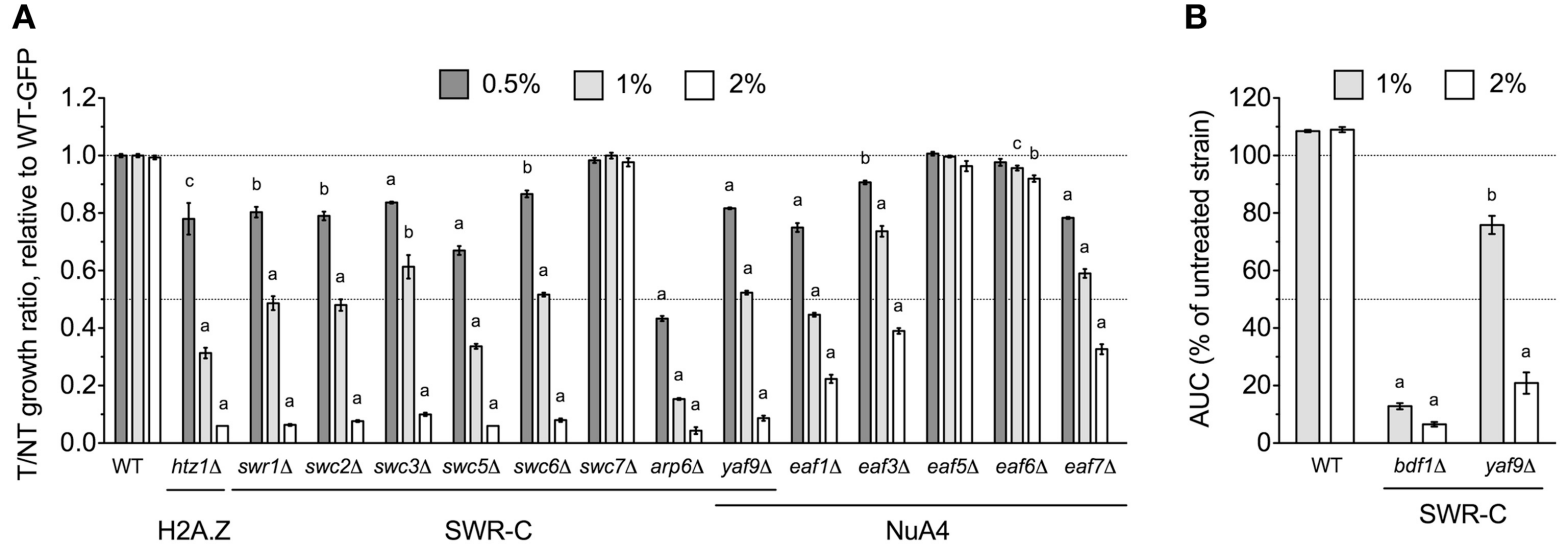
**SWR1 histone exchange and NuA4 histone H4 acetyltransferase mutants are sensitive to DMSO**. Statistical significance between wild-type and mutant strains was determined by *t*-test, where ^a^*p* < 0.001, ^b^*p* < 0.01, and ^c^*p* < 0.05. **(A)** Assessment of DMSO treatment on strains lacking components of SWR1 or NuA4. Relative growth ratios were obtained for three independent cultures and analyzed as described in Materials and Methods. **(B)** Evaluation of the *bdf1*Δ and *yaf9*Δ SWR1 mutants in DMSO. Growth curves were acquired from three independent cultures at the indicated doses.

**Figure 3:**
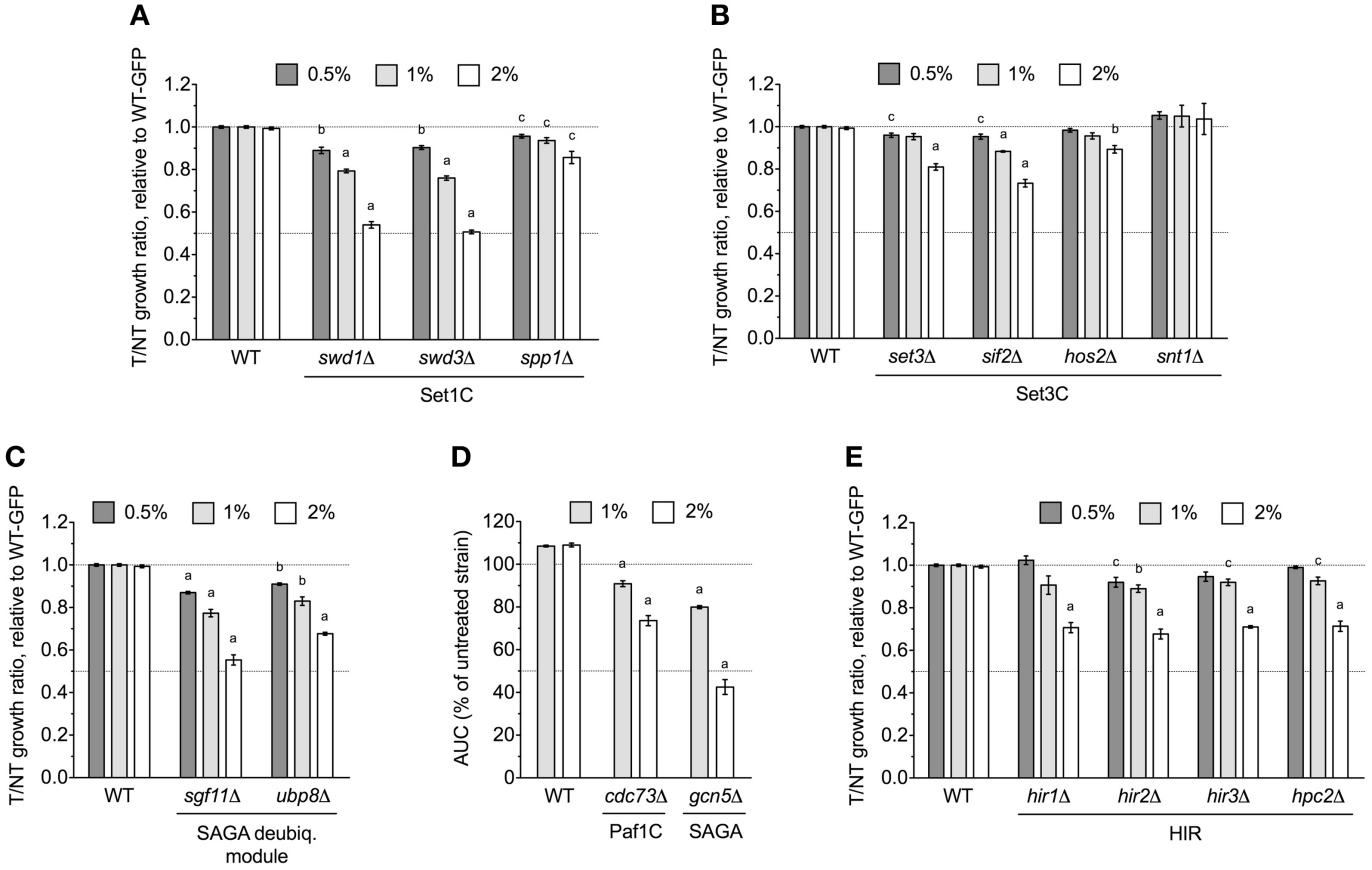
**Various chromatin remodeling mutants are sensitive to DMSO**. Statistical significance between wild-type and mutant strains was calculated by *t*-test, with ^a^*p* < 0.001, ^b^*p* < 0.01, and ^c^*p* < 0.05. **(A)** Relative growth assays for Set1C histone H3 methylase mutants in DMSO. **(B)** Relative growth assays for DMSO-treated Set3C histone deacetylase mutants. **(C)** Evaluation of DMSO treatment on strains lacking SAGA histone H2B deubiquitylation module components. **(D)** Growth curves for SAGA and Paf1 mutants in DMSO. **(E)** Relative growth experiments for DMSO-exposed HIR mutants. For **(A–C,E)**, relative growth ratios were obtained and averaged for three independent cultures, while **(D)** displays average area under the curve data for growth curves acquired from three cultures.

The *yaf9*Δ strain, which lacks a subunit common to the SWR1 histone exchange and NuA4 histone H4 acetyltransferase complexes, was identified by DSSA as DMSO-sensitive (Table 1) and confirmed by both competitive growth and growth curve assays to exhibit severe DMSO-dependent growth defects (Figures 2A,B). This stark phenotype prompted us to examine all non-essential SWR1 and NuA4 deletions for DMSO sensitivity, as SWR1 and NuA4 complexes cooperate to alter chromatin structure in yeast (reviewed by Lu et al., 2009). Except for *swc7*Δ, every SWR1 mutant (*swr1*Δ, *swc2*Δ, *swc3*Δ, *swc5*Δ, *swc6*Δ, *arp6*Δ, and *bdf1*Δ) was confirmed as sensitive to DMSO, with most displaying similar dose-dependent growth inhibition (Figures 2A,B). Moreover, *htz1*Δ, a strain lacking the histone variant H2A.Z exchanged for histone H2A in nucleosomes by the SWR1 complex (Mizuguchi et al., 2004), displayed growth defects in DMSO (Figure 2A). Several, but not all, non-essential NuA4 deletion mutants (*eaf1*Δ, *eaf3*Δ, and *eaf7*Δ, but not *eaf5*Δ or *eaf6*Δ) were DMSO-sensitive, however, levels of DMSO-mediated growth inhibition did not approach that of the SWR1 mutants (Figure 2A). We tested additional strains exhibiting both (1) defects in histone modification and (2) synthetic lethality or sickness with SWR1 and/or NuA4 genes (Collins et al., 2007; Mitchell et al., 2008; Costanzo et al., 2010; Hoppins et al., 2011). Absence of components of the Set1C histone H3 methylase (*swd1*Δ, *swd3*Δ, and *spp1*Δ), the Set3C histone deacetylase (*set3*Δ, *sif2*Δ, and *hos2*Δ, but not *snt1*Δ), the SAGA acetyltransferase (*gcn5*Δ) and histone H2B deubiquitylation module (*sgf11*Δ and *ubp8*Δ), and the Paf1 transcription initiation complex (*cdc73*Δ) conferred DMSO sensitivity, although none displayed DMSO-mediated growth defects as drastic as SWR1 mutants (Figures 3A–D). DSSA and our relative growth assay identified *HIR3*, a gene encoding a subunit of the histone regulation (HIR) nucleosome assembly complex, as required for DMSO tolerance, with additional HIR members (*HIR1, HIR2, HPC2*) also confirmed as necessary for resistance (Figure 3E).

### Additional mutants, including those involved in DNA repair, are sensitive to DMSO

**Figure 4:**
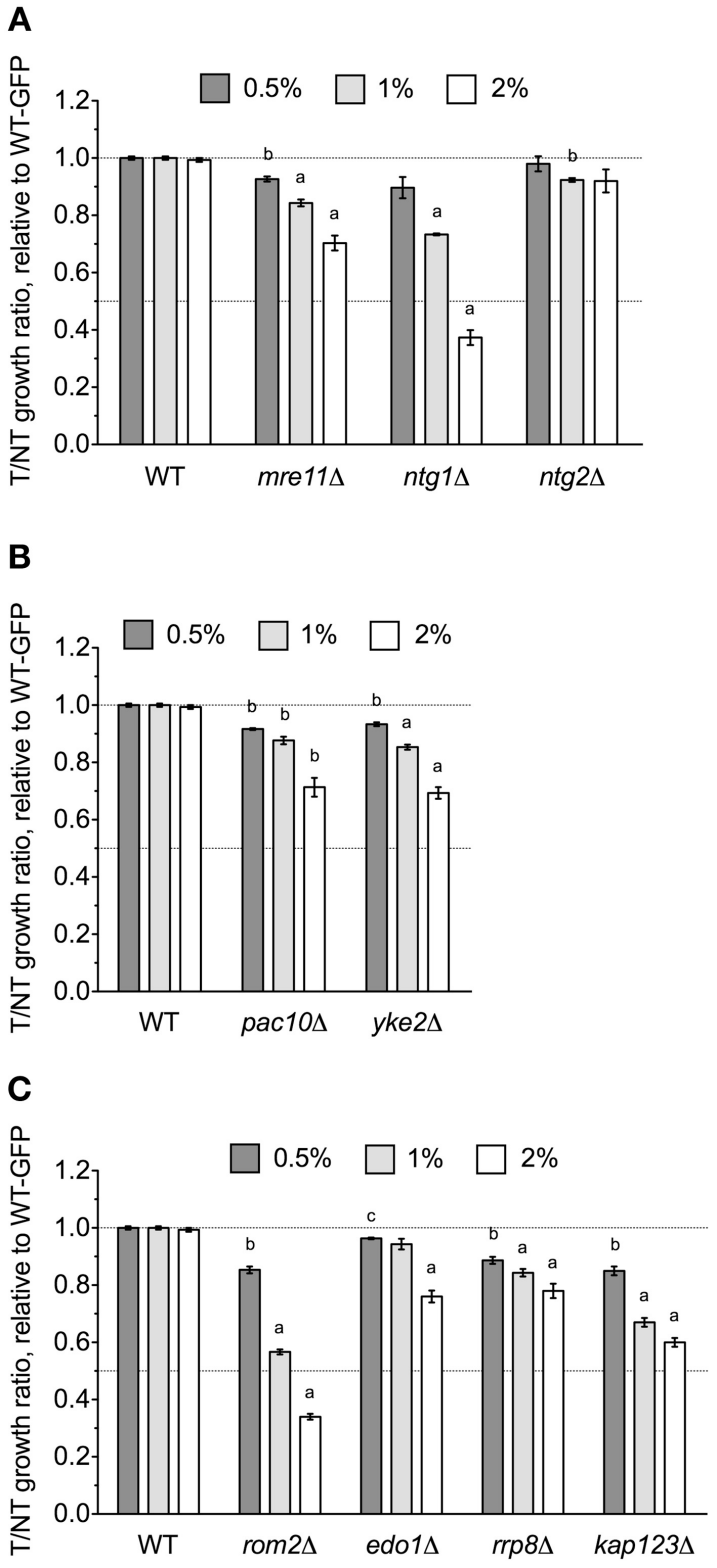
**DNA repair and other various mutants are sensitive to DMSO**. Relative growth assays were performed for three independent cultures. Ratio means and standard errors are shown, with statistical significance between wild-type and mutant strains calculated by *t*-test, where ^a^*p* < 0.001, ^b^*p* < 0.01, and ^c^*p* < 0.05. **(A)** Analysis of DNA repair mutant growth in DMSO. **(B)** Relative growth assays in DMSO with mutants lacking prefoldin components. **(C)** A summary of various additional mutants tested for sensitivity to DMSO.

The *NTG1* gene, which encodes a DNA N-glycosylase and apurinic/apyrimidinic lyase involved in base excision repair (Eide et al., 1996), was identified by DSSA as required for DMSO resistance (Table 1). Our relative growth assay confirmed *ntg1*Δ as sensitive to DMSO, but interestingly, deletion of the *NTG1* paralog *NTG2* did not markedly alter growth in DMSO (Figure 4A). A strain deleted for *MRE11*, a component of the meiotic recombination (MRX) complex involved in repair of DNA double-strand breaks (and exhibiting synthetic sickness with *EAF1* of NuA4), was also sensitive to DMSO (Figure 4A). Deletions in prefoldin (*pac10*Δ and *yke2*Δ), a complex involved in the folding of tubulin and actin, were sensitive to DMSO (Figure 4B). Other genes necessary for DMSO tolerance included *ROM2* (a GDP/GTP exchange factor for the Rho family), *EDO1* (of unknown function), *RRP8* (an rRNA methyltransferase), and *KAP123* (a nuclear importer of histones H3 and H4) (Figure 4C).

### Overexpression of H2A.Z confers resistance to DMSO

**Figure 5:**
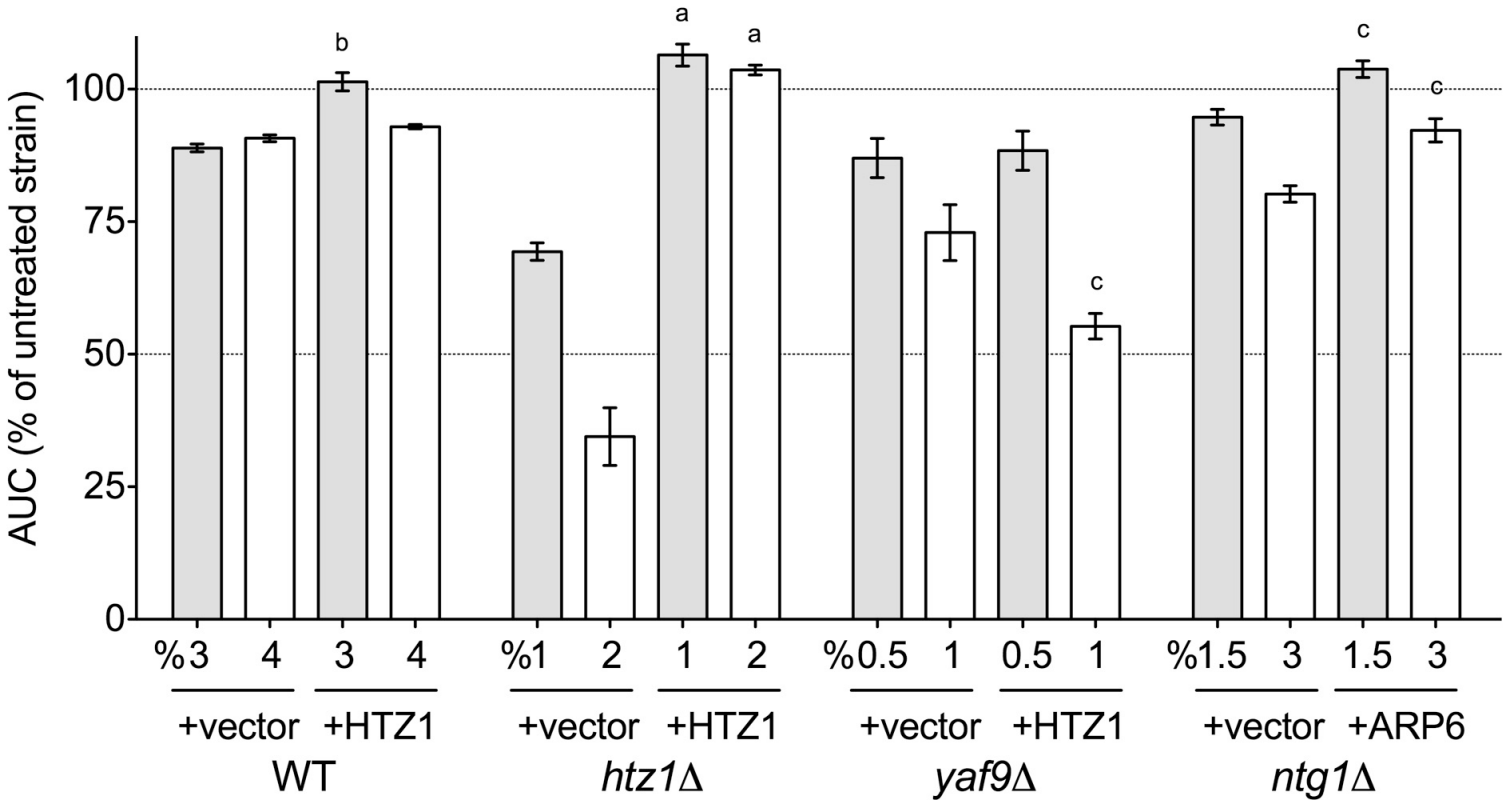
**Overexpression of Htz1p or Arp6p rescues DMSO sensitivity in various mutants**. Growth curves for three independent cultures were obtained in the indicated doses of DMSO. The area under the curve (AUC) means and standard error are shown. Statistical significance between AUCs for corresponding doses in the empty vector and overexpression strains was calculated by *t*-test, and is indicated by ^a^*p* < 0.001, ^b^*p* < 0.01, and ^c^*p* < 0.05.

After demonstrating a role for the SWR1 histone exchange machinery and its accessories in DMSO tolerance (Figure 2), we examined whether overexpression of Htz1p (histone H2A.Z exchanged for H2A by SWR1) or Arp6p (the nucleosome binding component of SWR1) could rescue the DMSO sensitivity of various strains. Increased levels of Htz1p reversed the DMSO sensitivity of BY4743 wild-type and *htz1*Δ, but interestingly, caused growth defects with 1% DMSO in the *yaf9*Δ strain (Figure 5). It did not affect sensitivity of the *ntg1*Δ DNA repair mutant (data not shown). Although Arp6p overexpression provided DMSO resistance to the *ntg1*Δ mutant (Figure 5), it did not alter the growth of wild-type, *htz1*Δ, or *yaf9*Δ strains in DMSO (data not shown).

## Discussion

DMSO is a polar and aprotic solvent commonly utilized to solubilize chemicals during toxicological or pharmaceutical inquiries (Santos et al., 2003). Compared to other solvents within its class such as sulfolane, *N,N*-dimethylformamide, *N*-methyl-pyrrolidin-2-one, or *N,N*-dimethyl acetamide, DMSO exhibits relatively limited acute toxicity (Tilstam, 2012), thus affording it preferred status within these fields. Despite its universality, DMSO's molecular mechanism(s) of action remain ambiguous, thus requiring investigations into the cellular processes and pathways it may perturb. Here we conducted a genome-wide functional screen in the model eukaryote *S. cerevisiae* to identify the non-essential yeast deletion mutants experiencing growth defects in 1% DMSO, a concentration typical to yeast toxicant or drug profiling studies. We demonstrate that components of the COG Golgi/ER transport and SWR1 histone exchange complexes are required for DMSO tolerance in yeast, with various mutants displaying sensitivity at concentrations as low as 0.25% (Figures 1, 2). Although many DMSO resistance genes are conserved in humans (Table 3), we were unable to confirm a role in DMSO tolerance for the *COG5, NTG1*, and *YAF9* homologs in the nematode *Caenorhabditis elegans* or the *COG7* and *COG8* homologs in human fibroblasts (data not shown). These results may indicate that DMSO's mechanism of toxicity in yeast is different from that exhibited in nematodes or human cells. However, if the toxic mechanism remains similar, it is feasible that compensatory cellular processes or genes are present in these mutants.

**Table 3:** **Human orthologs of yeast genes required for DMSO tolerance**.

**Yeast gene**	**Human ortholog(s)**	**Human protein description**
*ARP6*	*ACTR6*	ARP6 actin-related protein 6 homolog
*BDF1*	*EP300*	Histone acetyltransferase
*CDC73*	*CDC73*	Component of the PAF1 complex; tumor suppressor
*COG5*	*COG5*	Component of oligomeric Golgi complex 5
*COG6*	*COG6*	Component of oligomeric Golgi complex 6
*COG7*	*COG7*	Component of oligomeric Golgi complex 7
*COG8*	*COG8*	component of oligomeric Golgi complex 8
*EAF3*	*MORF4L1*	Component of the NuA4 histone acetyltransferase complex
*EAF6*	*MEAF6*	Component of the NuA4 histone acetyltransferase complex
*EAF7*	*MRGBP*	Component of the NuA4 histone acetyltransferase complex
*GCN5*	*KAT2A*	Histone acetyltransferase
*GOS1*	*GOSR1*	Involved in ER-Golgi transport as well as intra-Golgi transport
*HIR1/2*	*HIRA*	Histone chaperone
*HOS2*	*HDAC3*	Histone deacetylase
*HTZ1*	*H2AFZ*	Variant histone H2A; replaces conventional H2A in a subset of nucleosomes
*KAP123*	*IPO4*	Nuclear transport receptor
*MRE11*	*MRE11A*	Component of MRN complex; involved in DNA double-strand break repair
*NTG1*	*NTHL1*	Apurinic and/or apyrimidinic endonuclease and DNA N-glycosylase
*PAC10*	*VBP1*	Transfers target proteins to cytosolic chaperonin
*RRP8*	*RRP8*	Component of the eNoSC complex; mediates silencing of rDNA
*SIF2*	*TBL1X*	Subunit in corepressor SMRT complex along with HDAC3
*SPP1*	*CXXC1*	Recognizes CpG sequences and regulates gene expression
*SWC2*	*VPS72*	subunit of acetyltransferase TRRAP/TIP60 and chromatin-remodeling SRCAP
*SWC5*	*CFDP1*	Craniofacial development protein 1; may play role in embryogenesis
*SWC6*	*ZNHIT1*	Zinc finger, HIT-type containing 1
*SWD1*	*RBBP5*	Component of MLL1/MLL histone methyltransferase complex
*SWD3*	*WDR5*	Component of MLL1/MLL histone methyltransferase complex
*SWR1*	*SRCAP*	Catalytic component of the chromatin-remodeling SRCAP complex
*UBP8*	*USP22*	Histone deubiquitinating component of SAGA histone acetylation complex
*VAM7*	*SNAP25*	t-SNARE involved in the molecular regulation of neurotransmitter release
*VPS51*	*VPS51*	Required for both Golgi structure and vesicular trafficking
*VPS54*	*VPS54*	Required for retrograde transport of proteins from prevacuoles to the late Golgi
*YAF9*	*YEATS4*	Component of the NuA4 histone acetyltransferase complex
*YKE2*	*PFDN6*	Subunit of heteromeric prefoldin; transfers proteins to cytosolic chaperonin

Deletion of the yeast genes listed resulted in sensitivity to DMSO (shown in alphabetical order).

During the preparation of this manuscript, a report was published describing functional profiling of yeast mutants in DMSO (Zhang et al., 2013), with findings congruent to those presented in this study (see Table 4 for a comparison of strains identified). In this section, we discuss various aspects differentiating our investigation from Zhang et al. (2013). First, while these researchers assessed growth of individual yeast mutants via colony size on solid media, we performed functional profiling in pooled liquid cultures under competitive growth conditions. Our analyses, in which DNA sequences unique to each strain are hybridized to a microarray after toxicant exposure, are able to discern small growth defects and can identify sensitive strains overlooked by other methods (Table 4). However, the stringency of our DSSA may hinder identification of slow growing strains or those close to background levels. Nevertheless, these data are extremely relevant to those conducting pooled growth assays, especially considering the increased popularity of automated screens and high-throughput multiplexed barcode sequencing to examine strain growth in DMSO-soluble toxicants or drugs (Smith et al., 2010, 2012). Second, compared to the use of 4 and 8% DMSO in Zhang et al. (2013), the concentrations utilized in our screen (1%) and confirmation assays (0.25–2%) do not inhibit growth of the BY4743 wild-type strain and represent levels standard to functional screens (1% or less). The contrasting choice of doses may also account for differences in the DMSO-sensitive strains identified by each screen. Third, we provide extensive DMSO dose-response analyses for novel DMSO-sensitive strains as well as those concomitantly identified by Zhang et al. (2013). Finally, our overexpression data demonstrates that increased levels of Htz1p or Arp6p can rescue the growth of various deletion strains in DMSO (Figure 5).

**Table 4:** **A comparison between studies identifying yeast genes responsible for DMSO tolerance**.

**DMSO tolerance genes identified by Zhang et al. (2013) and this study**		**DMSO tolerance genes identified by this study**		
*ARP6*	*ROM2*	*COG5*	*KAP123*	*UBC8*
*BDF1*	*SET3*	*COG8*	*MRE11*	*VPS54*
*CDC73*	*SWC2 (VPS72)*	*EAF6*	*NTG1*	*YAF9*
*COG6*	*SWC3*	*EAF7*	*PAC10*	*YKE2*
*COG7*	*SWC6 (VPS71)*	*EDO1*	*RIC1*	
*EAF1*	*SWC7*	*GCN5*	*RRP8*	
*EAF3*	*SWD1*	*GET1*	*SGF11*	
*GOS1*	*SWR1*	*GET2*	*SIF2*	
*HIR2*	*VAM7*	*HIR1*	*SPP1*	
*HOS2*	*VPS51*	*HIR3*	*SWC5*	
*HTZ1*		*HPC2*	*SWD3*	

DMSO tolerance genes identified by Zhang et al. (2013) were compared to those identified in this study.

We have identified three cellular processes influencing DMSO resistance in budding yeast: Golgi/ER trafficking, SWR1 complex action, and DNA repair. Microarray analyses assessing the response of *S. cerevisiae* to DMSO (Zhang et al., 2003) did not identify any genes described in this study, however, correlation between transcriptional events and genes required for growth under a selective condition is often low (Giaever et al., 2002). The requirement of COG and SNARE Golgi/ER genes for DMSO tolerance (Figure 1) may reflect findings in human and rat hepatocytes, where DMSO altered expression of genes associated with SNARE interactions in vesicular transport (Sumida et al., 2011). Furthermore, as a “chemical chaperone,” DMSO can mimic the function of molecular chaperones (Papp and Csermely, 2006), a group of proteins closely tied to Golgi/ER operations. The DMSO sensitivity of histone H2A.Z and chromatin remodeling mutants (Figures 2, 3) indicate DMSO may affect chromatin structure. Lapeyre and Bekhor (1974) reported that 1% DMSO decreased chromatin thermostability, while higher concentrations promoted chromatin relaxation. Consistent with these findings, Pommier et al. (1983) suggested DMSO increased domain (loop) size by reducing DNA-protein attachment points after finding it enhanced intercalator-induced DNA breakage. DMSO could conceivably cause DNA damage, as demonstrated by DNA repair mutant sensitivity (Figure 4A). DMSO damaged DNA in bull sperm (Taşdemir et al., 2013) and erythroleukemic cells (Scher and Friend, 1978), and additionally altered expression of DNA repair genes in human and rat hepatocytes (Sumida et al., 2011).

The experimental evidence integrating the seemingly discrete processes of Golgi/ER transport, SWR1 complex action, and DNA repair is limited. Strains lacking SWR1 and NuA4 components exhibit synthetic lethality or sickness with various Golgi/ER transport and DNA repair genes (Collins et al., 2007; Mitchell et al., 2008; Costanzo et al., 2010; Hoppins et al., 2011), but mechanistic data explaining these findings are lacking. If Golgi/ER transport is the crucial determinant of DMSO tolerance, it is reasonable that loss of SWR1, which may repress transcription by preventing histone H2A.Z deposition into chromatin (Meneghini et al., 2003; Zhang et al., 2005), could confer DMSO sensitivity by decreasing production of Golgi/ER transport genes. Expression of COG7, a COG member involved in Golgi/ER trafficking, is downregulated in *htz1*Δ and the SWR1 mutants *swr1*Δ, *swc2*Δ, and *swc5*Δ (Morillo-Huesca et al., 2010), but others report the nonessential COG genes are neither induced nor repressed in the *swr1*Δ background (Meneghini et al., 2003). Alternatively, if SWR1 or H2A.Z activity is the deciding factor in DMSO resistance, defective Golgi/ER transport could prevent appropriate processing and localization of SWR1 components or H2A.Z. However, the expression of Golgi/ER, chromatin remodeling, or DNA repair genes described herein are not altered in *htz1*Δ, and further, *HTZ1* expression is unchanged in SWR1 or NuA4 mutants (Meneghini et al., 2003; Lindstrom et al., 2006; Lenstra et al., 2011). The relationship of SWR1 to DNA repair is evidenced by its ability to cause genetic instability in the absence of H2A.Z (Morillo-Huesca et al., 2010) and also deposit H2A.Z at double-stranded DNA breaks (Kalocsay et al., 2009).

We provide valuable insight into the genetic requirements for DMSO tolerance by identifying three major cellular processes—Golgi/ER transport, SWR1 complex function, and DNA repair—as important in DMSO resistance in *S. cerevisiae*. To separate effects of DMSO from a compound of interest, it is crucial for future yeast profiling studies to recognize that various deletion strains may fall out of pooled cultures during treatment with DMSO-solubilized drugs or toxicants. Data gathered by our study can direct additional experimentation to decipher the cellular and molecular mechanisms of DMSO action.

### Conflict of interest statement

The authors declare that the research was conducted in the absence of any commercial or financial relationships that could be construed as a potential conflict of interest.
